# Deep6: Classification of Metatranscriptomic Sequences into Cellular Empires and Viral Realms Using Deep Learning Models

**DOI:** 10.1128/mra.01079-22

**Published:** 2023-01-18

**Authors:** Jan F. Finke, Colleen T.E. Kellogg, Curtis A. Suttle

**Affiliations:** a Hakai Institute, Heriot Bay, British Columbia, Canada; b Department of Earth, Ocean, and Atmospheric Sciences, The University of British Columbia, Vancouver, British Columbia, Canada; c Department of Microbiology and Immunology, The University of British Columbia, Vancouver, British Columbia, Canada; d Department of Botany, The University of British Columbia, Vancouver, British Columbia, Canada; e Institute for the Oceans and Fisheries, The University of British Columbia, Vancouver, British Columbia, Canada; Loyola University Chicago

## Abstract

Deep6 is a deep learning model that classifies metatranscriptomic sequences as short as 250 nucleotides into prokaryotes, eukaryotes, or one of the four viral realms, using a reference-independent and alignment-free approach. Average accuracies range from 0.87 to 0.97, depending on sequence length.

## ANNOUNCEMENT

Viruses play key roles in marine ecosystems (1), but studying viral diversity and activity remains challenging. Recently, metatranscriptomes are being used to simultaneously profile biological functions and taxonomic information of cellular and viral origin in the same samples (2). Machine learning tools such as DeepVirFinder (3), DeepMicrobeFinder (4), and VirSorter2 (5) were designed to compensate for incomplete viral reference databases. However, these tools are limited to specific virus-host systems or struggle with short sequences. Here, we present Deep6, a reference-independent and alignment-free deep learning model to predict sequences for all viral realms, optimized for marine, short-sequence metatranscriptomic data.

Similar to DeepVirFinder and DeepMicrobeFinder, Deep6 is a deep learning model, but it expands on their functionality by classifying sequences into six groups, i.e., prokaryotes, eukaryotes, or one of the four viral realms (*Duplodnaviria*, *Varidnaviria*, *Monodnaviria*, or *Riboviria*). Deep6 is a multiclass convolutional neural network (CNN) model, consisting of 500 convolutions, 525 dense layers, and a default kernel size of 10. Four different default models accommodate input sequences of 250 to 499 nucleotides (nt), 500 to 999 nt, 1,000 to 1,499 nt, and >1,500 nt to optimize model performance for short metatranscriptomic sequences. Cellular training data are compiled from reference coding sequences (CDSs) for common marine eukaryote and prokaryote taxa (6). Viral training data are compiled from the CDSs for all available reference genomes and neighbor genomes in the NCBI database for viral orders in the four viral realms. For model training, each group is represented by 50,000 to 130,000 randomly selected CDSs from the pool of reference data. Additionally, Deep6 can be readily retrained for custom models.

The Deep6 repository includes the default models, the sequence prediction script, and scripts for batch encoding data sets and training custom models. For prediction, contigs of various lengths in FASTA format can be used; longer contigs have greater prediction accuracy. The prediction script automatically encodes and calculates group scores, and the highest score indicates the likely classification. The provided R script processes prediction scores for downstream analysis. The batch encoding script encodes overlapping forward and reverse chunks from the training and validation CDSs. The custom training script feeds data into the CNN and saves the best model. Model performance is assessed by the area under the receiver operating characteristic curve (AUROC), the average accuracy and group precision, recall, and derived F1 scores.

**FIG 1:**
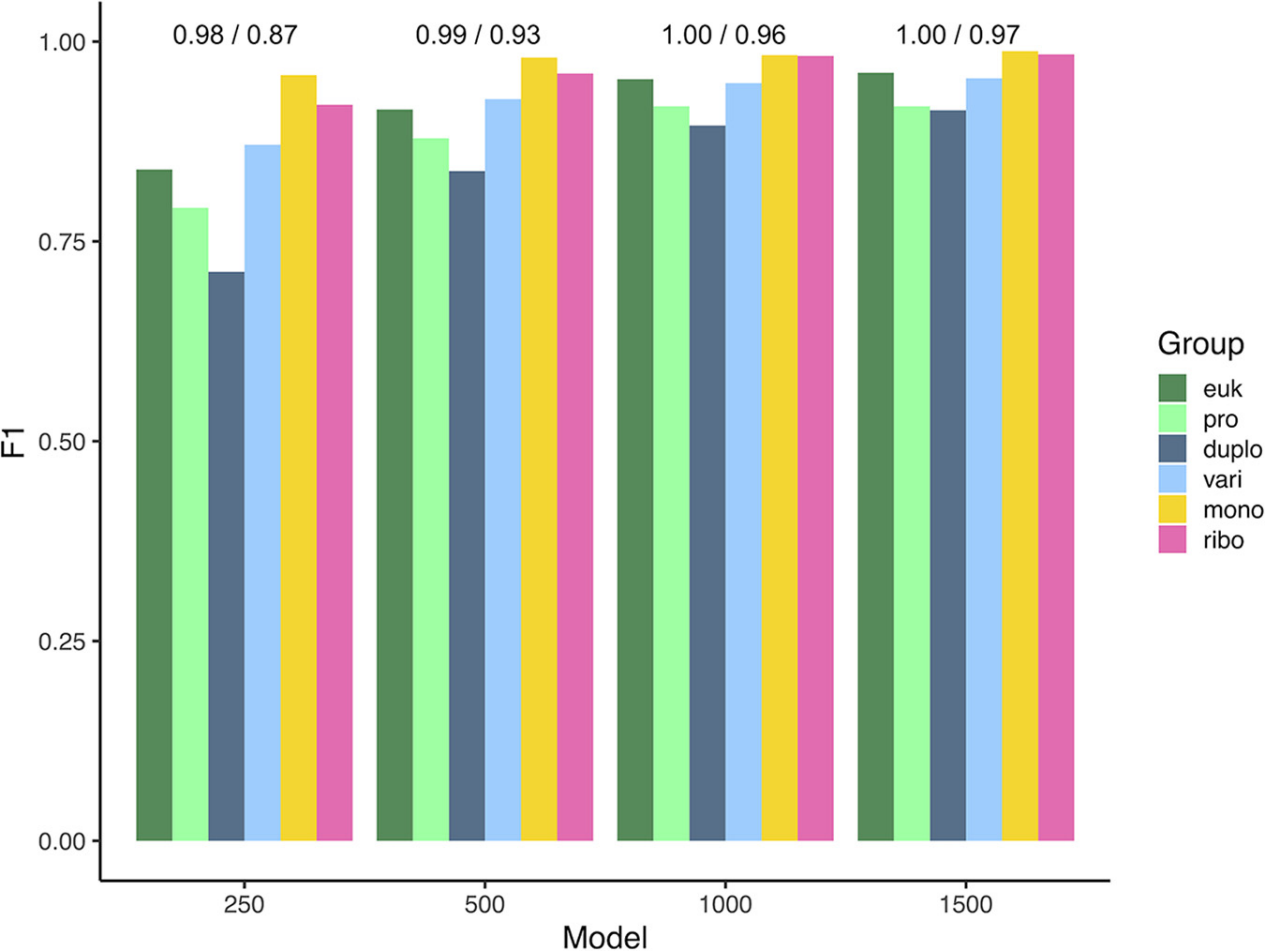
Validation F1 scores for the default models. Numbers of nucleotides are indicated, and the AUROC/average accuracy values are shown at the top. euk, eukaryotes; pro, prokaryotes; duplo, *Duplodnaviria*; vari, *Varidnaviria*; mono, *Monodnaviria*; ribo, *Riboviria*.

The default models have training and validation AUROCs ranging from 0.98 to 1.00 and average accuracies ranging from 0.87 to 0.97 (Fig. 1). Validation F1 scores ranged from 0.91 to 0.99 for the >1,500-nt model and from 0.71 to 0.96 for the 250-nt to 499-nt model. In a cross-validation with 30,000 CDSs, 80% of sequences were assigned to the correct group. F1 scores were as follows: eukaryotes, 0.79; prokaryotes, 0.83; *Duplodnaviria*, 0.65; *Varidnaviria*, 0.81; *Monodnaviria*, 0.87; *Riboviria*, 0.81. Where comparable, Deep6 matched or exceeded the performance of other tools. Deep6 is a high-throughput tool that can process about 100 thousand sequences per day on a single core, utilizing <1 GB of memory. Deep6 predicts viral sequences from metatranscriptomic data to support homology-based viral identification and discover novel viruses.

### Data availability.

Deep6 source code and supporting information are available (under a GNU Affero General Public License [AGPL] v3) at https://github.com/janfelix/Deep6. Scripts are written in Python3 and rely on the readily available libraries Biopython, Keras, Sklearn, and TensorFlow. Environmental files to set up Conda environments and install dependencies are also provided. Contact J.F.F. for further information on training and cross-validation data sets.
